# An interband cascade laser based heterodyne detector with integrated optical amplifier and local oscillator

**DOI:** 10.1515/nanoph-2023-0762

**Published:** 2024-02-05

**Authors:** Sandro Dal Cin, Andreas Windischhofer, Florian Pilat, Michael Leskowschek, Vito F. Pecile, Mauro David, Maximilian Beiser, Robert Weih, Johannes Koeth, Georg Marschick, Borislav Hinkov, Gottfried Strasser, Oliver H. Heckl, Benedikt Schwarz

**Affiliations:** Institute of Solid State Electronics, TU Wien, Gusshausstrasse 25-25a, 1040 Vienna, Austria; Faculty of Physics, Faculty Center for Nano Structure Research, Christian Doppler Laboratory for Mid-IR Spectroscopy, University of Vienna, Boltzmanngasse 5, 1090 Vienna, Austria; Vienna Doctoral School in Physics, University of Vienna, Boltzmanngasse 5, 1090 Vienna, Austria; Nanoplus Nanosystems and Technologies GmbH, Oberer Kirschberg 4, 97218 Gerbrunn, Germany

**Keywords:** interband cascade laser, heterodyne, racetrack, bi-functional ICL, detector, semicondutor optical amplifier

## Abstract

Heterodyne detection based on interband cascade lasers (ICL) has been demonstrated in a wide range of different applications. However, it is still often limited to bulky tabletop systems using individual components such as dual laser setups, beam shaping elements, and discrete detectors. In this work, a versatile integrated ICL platform is investigated for tackling this issue. A RF-optimized, two-section ICL approach is employed, consisting of a short section typically used for efficient modulation of the cavity field and a long gain section. Such a laser is operated in reversed mode, with the entire Fabry–Pérot waveguide utilized as a semiconductor optical amplifier (SOA) and the electrically separated short section as detector. Furthermore, a racetrack cavity is introduced as on-chip single-mode reference generator. The field of the racetrack cavity is coupled into the SOA waveguide via an 800 nm gap. By external injection of a single mode ICL operating at the appropriate wavelength, a heterodyne beating between the on-chip reference and the injected signal can be observed on the integrated detector section of the SOA-detector.

## 1 Introduction

ICLs were first proposed in 1995 [1] shortly after the first demonstration of the quantum cascade laser (QCL) [2]. Despite the prevalent focus on QCL research and development in recent years, ICLs offer a unique advantage in power consumption and thus integration by requiring fewer active region stages for lasing operation. As a result, especially the use of ICLs in their sweet-spot range at lower mid-infrared wavelengths becomes more attractive compared to QCLs, due to their lower lasing threshold [3]. Substantial progress has been achieved in recent years in understanding and optimizing ICLs [4], [5], enabling attainable wavelengths of 6 μm [6] in continuous wave operation at room temperature and up to 14 μm at cryogenic temperatures [7], [8]. The most prominent applications regarding both QCLs and ICLs can be linked to spectroscopy and sensing in the molecular fingerprint region [9]–[13] as well as free-space optical communication applications [14], [15].

Heterodyne- and multi-heterodyne detection [16], [17] in combination with bi-functional ICL material systems [18] hold great potential for research benefiting from the setup proposed in this work, especially in the light of miniaturization and on-chip integration, which often is a crucial requirement for on-site or even *in-situ* applications [19]. For efficient and sensitive real-time monitoring and analysis of specific molecules and compounds, single-mode heterodyne detection proves to be a promising approach [20]. We want to extend this concept, and utilize a RF-optimized [21], [22], two-section, bi-functional Fabry–Pérot ICL operated in reversed mode as combined SOA and detector setup for amplified detection. To enable this, the modulation-section is repurposed as detector and the entire ridge-waveguide gain-section as SOA. We further extend this approach in an additional proof-of-concept experiment by introducing a coupled racetrack resonator cavity section adjacent to the detector SOA-section. It is separated by an 800 nm gap and driven at low bias, thus, acting as an on chip single mode reference generator [23]. The reference and injected fields amplified by the SOA-section consequently produce a heterodyne beating signal in the detector-section.

## 2 Device characterization

### 2.1 Overview and methods

The manuscript is organized in the following way. First, we want to verify the basic operation principle of the SOA-detector system. Therefore, in Section 2.2 we investigate the amplification of the SOA-detector by using an identical device from the same process operated as laser for characterization. Subsequently, in Section 2.3 we evaluate the modal gain based on the experimental results for the amplification. With both, laser and detector, processed from the same bi-functional material, any insights obtained for the device operated as SOA-detector are inherently connected to the device in lasing operation. This enables the use of the methods described in Sections 2.2 and 2.3 for general material performance characterization. Finally, in Section 2.4 we investigate the RF-capabilities of the SOA-detector by means of the impulse response induced by femto-second pulses.

### 2.2 SOA-detector amplification

**Figure 1:**
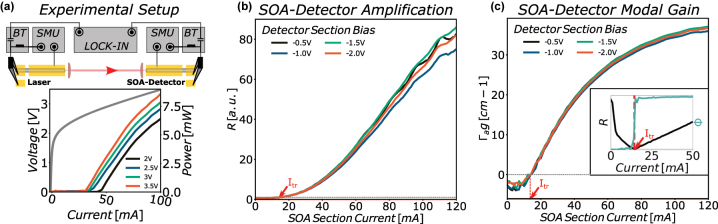
Detector characterization: (a) experimental setup for (b) and (c). Both laser- and detector-sections are biased with a SMU. The short sections are contacted with RF-probes and a bias-tee (BT). A lock-in amplifier is used to superimpose a modulation signal onto the DC bias of the short modulation-section of the laser. The signal is subsequently demodulated on the detector-section of the SOA-detector. Inset: The LIV characteristics of the laser recorded with a power meter for different bias voltages between 2 V and 3.5 V of the short section. (b) Evaluation of the SOA-detector amplification for different detector-section bias voltages. (c) Evaluation of the SOA-detector modal gain for different detector-section bias voltages. Inset: Experimental result for the transparency current of the SOA-section. Both plots (b) and (c) are normalized to the resulting transparency current of 14.4 mA.

The amplification of the SOA-detector is evaluated with the experimental setup illustrated in Figure 1(a). Both detector and laser, used for this initial characterization, are identical two-section Fabry–Pérot devices consisting of a 1200 μm long gain/SOA-section and a 300 μm long modulation/detector section electrically separated by an 800 nm gap. It is important to note here, that the separation between the two sections is limited to the sputtered gold contacting layer and the Fabry–Pérot waveguide itself is uninterrupted. For initial proof-of-concept, this combination of identical material systems is ideal because of the resulting spectral overlap between the two devices. The contacts of the short sections are optimized for RF operation and RF-probe contacting with minimized gold contact pads, with a size of 50 μm × 50 μm. For further increasing the RC-cut-off of the short sections, a 1200 nm thick SiN passivation layer is implemented below the contact pads. Both sections of the laser and the SOA-detector are biased using a source measure unit (SMU). A Bias-Tee is connected to both of the short sections providing the ability to superimpose a modulation signal on the DC bias of the modulation-section of the laser and demodulate the corresponding signal via a Lock-In amplifier at the detector-section of the SOA-detector. A positive bias is applied to the gain- and modulation-section of the laser employed for characterization and the corresponding LIV characteristic for different modulation-section biases is shown in the inset in Figure 1(a). The SOA-section is also operated under positive bias, while the detector-section is biased negatively. The most widespread type of SOA is the travelling wave amplifier, defined by extremely low facet reflectivities. This ensures that light coupled into the amplifier does not experience reflections inside the cavity and amplification and re-emission at the back facet occur during one single pass through the active material. The second category of SOA is the Fabry–Pérot (FP) SOA. FP-SOAs by means of predetermined and well defined facet reflectivities enable multiple passes of the signal to be amplified inside the amplifier cavity. This facilitates higher achievable amplification values at the expense of narrowband gain due to the wavelength selectivity determined by the cavity resonances [24], [25]. In our case, the negative bias of the detector-section is crucial to avoid multiple passes of the injected light in the cavity of the SOA-detector and thus operation of the detector as FP-SOA. Single pass operation suppresses the formation of Fabry–Pérot fringes due to gain saturation and facilitates traveling wave SOA operation without the requirement for AR coatings. As mentioned above, we can analyze the light-amplification in the SOA-section by superimposing an AC signal on the DC bias of the modulation-section of the laser and subsequently demodulating the resulting amplified signal at the detector-section of the SOA-detector. The SOA-section bias is swept, while the bias of both sections of the laser is kept constant for each measurement. The demodulated signal is recorded over a bias range of the SOA-section ranging from 0 mA up to 120 mA for different detector-section bias voltages. The Lock-In modulation signal amplitude for this measurement is set to 500 mV and the modulation frequency is set significantly above the lower cut-off frequency of the Bias-Tee (modulation signal frequency of 700 kHz for a Bias-Tee with lower cutoff of 20 kHz). Demodulation of the signal at the detector-section with a lock-in amplifier yields the amplitude *R* and phase Φ of the amplified input signal depending on the bias of the SOA-section. For evaluation of the amplification and the modal gain in Section 2.3, the recorded amplitude curve *R* has to be normalized at the material transparency current. Therefore, the detector-section of the SOA-detector is left unbiased and the Bias-Tee is connected to the SOA-section. The laser power is again kept constant and the transparency current is evaluated by locating the position of the sign flip in the amplitude characteristic *R* together with a clear phase jump in the phase characteristic Φ of the demodulated signal. Both, sign flip and corresponding phase jump, indicate the transition between absorption and amplification. The result is added as inset in Figure 1(c). The obtained transparency current of the SOA-section is 14.4 mA. We can now normalize the amplitude curve to obtain an amplification of 1 at the transparency current. The resulting normalized SOA-detector amplification characteristic is shown in Figure 1(b) indicating achievable amplification of the injected light from the laser of up to a factor of 85. An amplification factor of 85 in terms of injected and amplified optical power would correspond to a gain of 19.3 dB over the length of the SOA-section even without AR coatings. This result is in the same order of magnitude as more established reported SOA devices [26], [27], [28].

### 2.3 SOA-detecor modal gain

Based on the results in Section 2.2, we can access the modal gain of the SOA-section. Assuming a single pass of the injected light through the SOA-section, the change in optical power can be described by $P\left(L\right)=e^{\Gamma_{a}gL}$ with: *P*(*L*) the measured optical power, normalized to the transparency current at the detector-section, *L* the length of the SOA-section and Γ_*a*_ *g* the modal gain. *P*(*L*) is shown in Figure 1(b) enabling extraction of the modal gain by Γ_*a*_ *g* = ln(*P*(*L*))/*L*, with the results displayed in Figure 1(c).

### 2.4 SOA-detector impulse response

**Figure 2:**
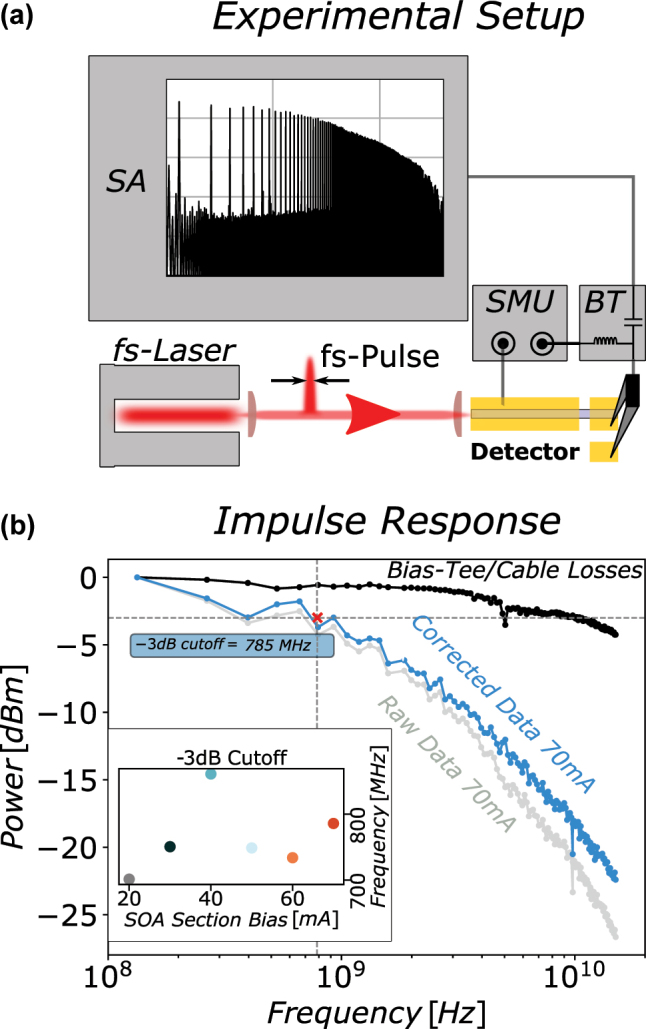
Impulse response: (a) Experimental setup for (b). A fs-pulses are coupled into the front facet of the SOA-detector. Both sections of the SOA-detector are biased with a SMU. The impulse response of the detector-section is accessed via a Bias-Tee on a spectrum analyzer (SA). (b) Exemplary detector impulse response evaluation for a SOA-section bias of 70 mA. A peak finding algorithm is applied to the SA trace to obtain the raw data (grey curve). The curve is then corrected for cable/Bias-Tee losses (black curve) providing the corrected data used for evaluation of the −3dB cutoff (blue curve). Inset: Evaluation as in (b) for SOA-section biases from 20 mA to 70 mA.

To demonstrate the capabilities for potential optical communication applications, we investigate the RF-capabilities of the SOA-detector in this section. The corresponding setup is illustrated in Figure 2(a). The SOA-detector is again biased with a SMU and the RF-signal on the short detector-section is accessed via a Bias-Tee with an upper cut-off frequency of 20 GHz. Femto-second pulses generated in an optical parametric oscillator (OPO) with a center wavelength of 3.3 μm and a repetition rate of 134.4 MHz are coupled into the SOA-detector and the FFT of the resulting impulse response [29] of the system is captured with a spectrum analyzer for different SOA-section biases between 20 mA and 70 mA. The SOA-detector spectrum evaluated in laser operation shows a reasonably well matched center wavelength located at 3.34 μm. The corresponding spectrum of the SOA-detector operated in lasing mode is given in the Supplementary Material. The plot given in Figure 2(b) shows an overview of the evaluation process for the recorded data for the highest SOA-section bias of 70 mA and a detector-section bias of −2.0 V. After applying a peak finding algorithm on the spectrum analyzer data, the raw data is normalized (grey curve) and corrected for the losses introduced by the cables and the Bias-Tee (black curve). Subsequently to the correction for the losses (blue curve), the −3dB cutoff of the SOA-detector was estimated to 785 MHz. The inset in Figure 2(b) shows the evaluation of the −3dB cutoffs for the recorded impulse responses, displaying results between 700 MHz and 840 MHz for the different SOA-section biases. We can conclude, that the −3dB cutoff is almost independent of the applied SOA-section bias. The achievable speed in this configuration is enabled by the rather small contact pads and the increased SiN passivation thickness, paired with the use of RF-probes for contacting.

## 3 Application

**Figure 3:**
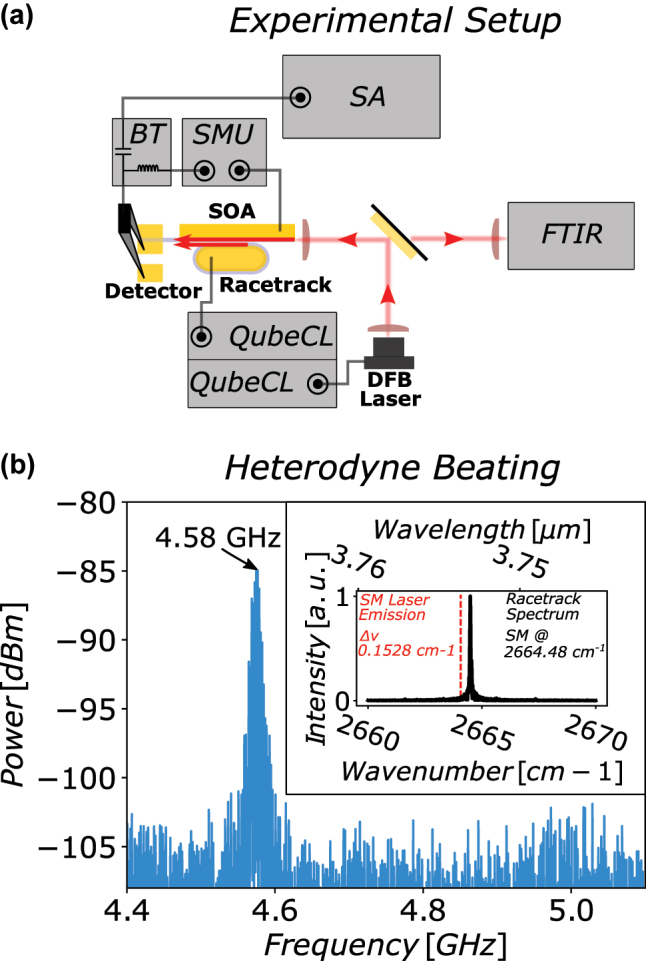
Heterodyne beating: (a) Experimental setup for heterodyne beating measurement. The racetrack section is driven with a QubeCL low-noise current driver ensuring single-mode operation with an FTIR accessible via flip mirror. The signal is coupled into the SOA-detector via an 800 nm gap. A DFB laser is then injected into the front facet of the SOA-detector. Both signals are amplified and superimposed on the detector-section. The SOA-detector is biased with a SMU and the RF signal is accessed via a Bias-Tee. (b) Spectrum analyzer trace of the heterodyne beating between the racetrack mode and the DFB laser mode at 4.6 GHz. Inset: Single mode emission spectrum of the racetrack recorded with the FTIR. The DFB laser emission frequency is indicated with a red line.

For demonstrating the maturity of the investigated approach, a possible application for the SOA-detector is illustrated in Figure 3(a). A racetrack cavity was added adjacent to the SOA-detector [23], with circumference of 1830 μm and a coupling region length of 600 μm separated from the SOA-waveguide by an 800 nm gap. The detector-section length for this device is 200 μm and the SOA-section length was increased to 4000 μm. For the experiment, the racetrack section is driven with a ppqSense QubeCL low noise current driver at a low bias of 44 mA/3.95 V and single-mode operation was validated with an FTIR. The resulting spectrum of the racetrack cavity is shown in the inset of Figure 3(b), demonstrating single mode operation at 2664.48 cm^−1^. Both, the detector- and SOA-section are biased with a SMU with a fixed SOA-section bias of 95.9 mA/3.9 which was yielding the best results. To achieve a heterodyne beating on the detector-section of the SOA-detector, a DFB single-mode laser from nanoplus driven with a second low noise current driver is injected into the SOA-detector. The latter was spectrally tuned to emit very close to the racetrack cavity mode at Δ*ν* = 0.1528 cm^−1^. In this configuration, a pronounced beating between the racetrack mode and the injected single mode laser could be observed on the detector-section for a large detector bias range from −0.2 V up to −2.0 V, using a Bias-Tee and Spectrum Analyzer with resolution bandwidth set to 110 kHz for analysis. By tuning the temperature of the single-mode laser, the beating signal on the spectrum analyzer could be shifted and measured with the on-chip detector in a range from 100 MHz up to 20 GHz [see Supplementary Information]. Successful implementation of such a design in future applications strongly relies on achieving low frequency noise operation for the entire measurement setup. Even though the intrinsic linewidth of ICLs was reported as low as 10 kHz [30], [31], [32], in real world applications ICLs are highly susceptible to current and temperature fluctuations and observed linewidths in the range of several hundred kHz to MHz are not uncommon [33]. In the light of increase in sensitivity and applicability of the presented proof-of-concept, the employment of ultra-low noise current driving electronics and temperature stabilization in concealed atmosphere will be imperative.

## 4 Conclusions

In conclusion, we presented a promising platform for on-chip heterodyne detection based on a two-section bi-functional Fabry–Pérot ICL, operated in reverse as SOA-detector in combination with a racetrack cavity as single mode local oscillator. We first evaluated the gain of a standalone SOA-detector utilizing the same device operated as laser injected into the SOA-detector for characterization. We demonstrate amplification up to a factor of 85, translating to a modal gain above 30 cm^−1^ without the need for application of AR coatings. Additionally, by measuring the impulse response of the detector-section with femto-second pulses generated with an OPO, we demonstrated −3 dB RF-cut-off frequencies of the SOA-detector of up to 785 MHz. Finally, we have shown a proof-of-concept measurement on the SOA-detector, recording the heterodyne beating signal between an injected DFB single-mode ICL and the integrated single-mode racetrack cavity reference signal. The results presented in this study show great potential for future integrated optical communication and sensing applications on a platform that is based on a widely used two-section ICL without the requirement for additional processing steps.

## Supplementary Material

Supplementary Material Details

Supplementary Material Details

## References

[1] Yang R. Q. (1995). Infrared laser based on intersubband transitions in quantum wells. *Superlattices Microstruct.*.

[2] Faist J., Capasso F., Sivco D. L., Sirtori C., Hutchinson A. L., Cho A. Y. (1994). Quantum cascade laser. *Science*.

[3] Weih R., Kamp M., Höfling S. (2013). Interband cascade lasers with room temperature threshold current densities below 100 A/cm2. *Appl. Phys. Lett.*.

[4] Meyer J. (2020). The interband cascade laser. *Photonics*.

[5] Knötig H. (2022). Mitigating valence intersubband absorption in interband cascade lasers. *Laser Photonics Rev.*.

[6] Nauschütz J., Scheuermann J., Weih R., Koeth J., Schwarz B., Höfling S. (2023). Room temperature operation of single mode GaSb‐based DFB interband cascade lasers beyond 6.1 µm. *Electron. Lett.*.

[7] Massengale J. A., Shen Y., Yang R. Q., Hawkins S. D., Klem J. F. (2022). Long wavelength interband cascade lasers. *Appl. Phys. Lett.*.

[8] Shen Y., Massengale J. A., Yang R. Q., Hawkins S. D., Muhowski A. J. (2023). Pushing the performance limits of long wavelength interband cascade lasers using innovative quantum well active regions. *Appl. Phys. Lett.*.

[9] Yan G. (2022). Mobile vehicle measurement of urban atmospheric CH_4_/C_2_H_6_ using a midinfrared dual-gas sensor system based on interband cascade laser absorption spectroscopy. *IEEE Trans. Instrum. Meas.*.

[10] Gomółka G., Stȩpniewski G., Pysz D., Buczyński R., Klimczak M., Nikodem M. (2023). Highly sensitive methane detection using a mid-infrared interband cascade laser and an anti-resonant hollow-core fiber. *Opt. Express*.

[11] Stiefvater G., Hespos Y., Wiedenmann D., Lambrecht A., Brunner R., Wöllenstein J. (2023). A portable laser spectroscopic system for measuring nitrous oxide emissions on fertilized cropland. *Sensors*.

[12] Wysocki G. (2007). Dual interband cascade laser based trace-gas sensor for environmental monitoring. *Appl. Opt.*.

[13] Kazakov D. (2024). Active mid-infrared ring resonators. *Nat. Commun*.

[14] Spitz O. (2022). Free-space communication with directly modulated mid-infrared quantum cascade devices. *IEEE J. Sel. Top. Quantum Electron.*.

[15] Flannigan L., Yoell L., qing Xu C. (2022). Mid-wave and long-wave infrared transmitters and detectors for optical satellite communications—a review. *J. Opt.*.

[16] Sterczewski L. A., Bagheri M., Frez C., Canedy C. L., Vurgaftman I., Meyer J. R. (2020). Mid-infrared dual-comb spectroscopy with room-temperature bi-functional interband cascade lasers and detectors. *Appl. Phys. Lett.*.

[17] Shen F. (2021). Transportable mid-infrared laser heterodyne radiometer operating in the shot-noise dominated regime. *Opt. Lett.*.

[18] Schwarz B. (2019). Monolithic frequency comb platform based on interband cascade lasers and detectors. *Optica*.

[19] Meyer J. R. (2021). Interband cascade photonic integrated circuits on native III-V chip. *Sensors*.

[20] Levenson M. D., Eesley G. L. (1979). Polarization selective optical heterodyne detection for dramatically improved sensitivity in laser spectroscopy. *Appl. Phys.*.

[21] Piccardo M. (2018). Time-dependent population inversion gratings in laser frequency combs. *Optica*.

[22] Hillbrand J. (2020). Mode-locked short pulses from an 8 μm wavelength semiconductor laser. *Nat. Commun.*.

[23] Bewley W. W. (2011). *CLEO:2011 – Laser Applications to Photonic Applications*.

[24] Jiang F., Yu Y., Cao T., Tang H., Dong J., Zhang X. (2016). Flat-top bandpass microwave photonic filter with tunable bandwidth and center frequency based on a Fabry–Pérot semiconductor optical amplifier. *Opt. Lett.*.

[25] Olsson N. (1992). Semiconductor optical amplifiers. *Proc. IEEE*.

[26] Davenport M. L., Skendzic S., Volet N., Hulme J. C., Heck M. J. R., Bowers J. E. (2016). Heterogeneous silicon/III–V semiconductor optical amplifiers. *IEEE J. Sel. Top. Quantum Electron.*.

[27] Akiyama T., Sugawara M., Arakawa Y. (2007). Quantum-dot semiconductor optical amplifiers. *Proc. IEEE*.

[28] Sobhanan A. (2022). Semiconductor optical amplifiers: recent advances and applications. *Adv. Opt. Photonics*.

[29] Hillbrand J. (2021). High-speed quantum cascade detector characterized with a mid-infrared femtosecond oscillator. *Opt. Express*.

[30] Borri S., Siciliani de Cumis M., Viciani S., D’Amato F., De Natale P. (2020). Unveiling quantum-limited operation of interband cascade lasers. *APL Photonics*.

[31] Du Z., Luo G., An Y., Li J. (2016). Dynamic spectral characteristics measurement of DFB interband cascade laser under injection current tuning. *Appl. Phys. Lett.*.

[32] Li X.-Y., Fan Z.-F., Deng Y., Wang C. (2022). 30-kHz linewidth interband cascade laser with optical feedback. *Appl. Phys. Lett.*.

[33] Deng Y., Zhao B.-B., Wang X.-G., Wang C. (2020). Narrow linewidth characteristics of interband cascade lasers. *Appl. Phys. Lett.*.

