# Supplementary figures and images for: An interband cascade laser based heterodyne detector with integrated optical amplifier and local oscillator
Source: Nanophotonics. 2024 Feb 5;13(10):1759–64. doi: 10.1515/nanoph-2023-0762 (PMC11052533; doi:10.1515/nanoph-2023-0762)

**a**

Detector Section Bias: -0.2 V  
*Heterodyne Beating*

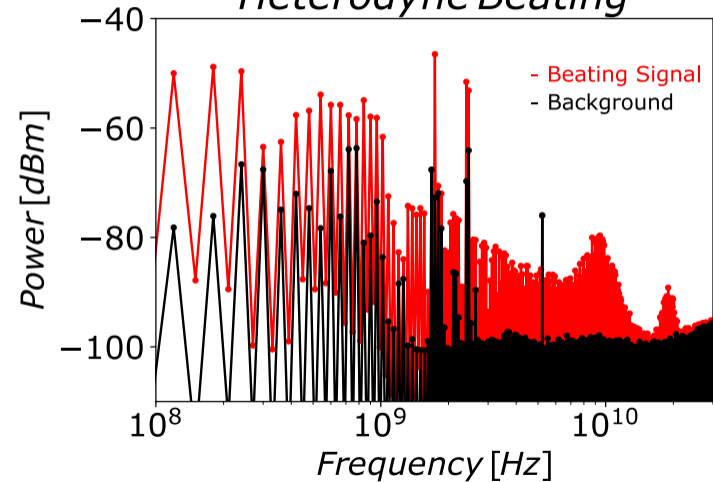**b**

Detector Section Bias: -2.0 V  
*Heterodyne Beating*

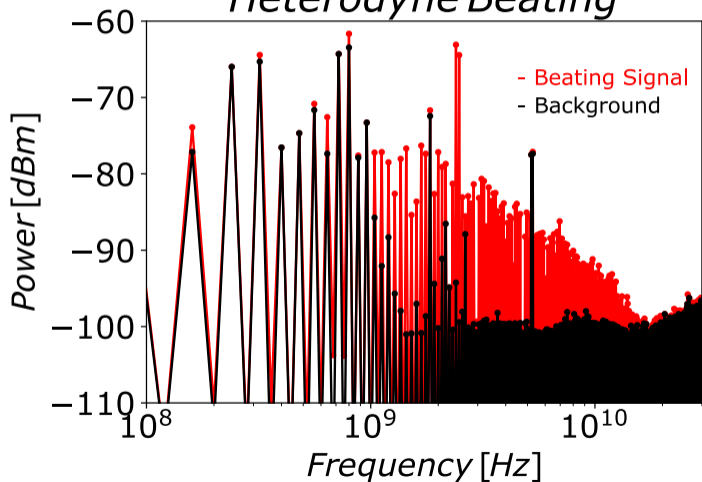

Supplement: Supplementary file 1 — Supplementary Material Details [file j_nanoph-2023-0762_suppl_001.zip › fig_1_supp.pdf]
