# Supplementary material for: An interband cascade laser based heterodyne detector with integrated optical amplifier and local oscillator
Source: Nanophotonics. 2024 Feb 5;13(10):1759–64. doi: 10.1515/nanoph-2023-0762 (PMC11052533; doi:10.1515/nanoph-2023-0762)
Supplement: Supplementary file 2 — Supplementary Material Details [file j_nanoph-2023-0762_suppl_002.pdf]

Supplementary Information: An interband cascade laser based heterodyne detector with integrated optical amplifier and local oscillator

Sandro Dal Cin,* Andreas Windischhofer, Florian Pilat, Michael Leskowschek, Vito F. Pecile, Mauro David, Maximilian Beiser, Robert Weih, Johannes Koeth, Georg Marschick, Borislav Hinkov, Gottfried Strasser, Oliver H. Heckl, and Benedikt Schwarz†

* sandro.cin@tuwien.ac.at

† benedikt.schwarz@tuwien.ac.at

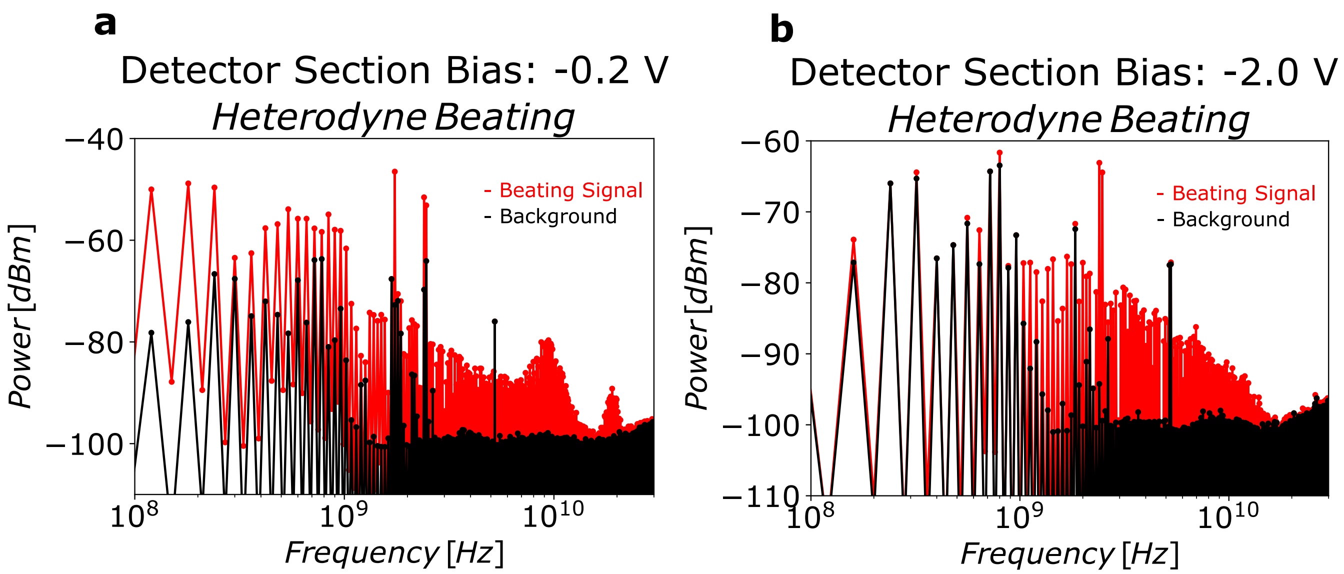

FIG. 1. **Supplemental Figure 1:** (a) Heterodyne beating between the externally injected single-mode laser and the on-chip racetrack signal recorded up to a frequency of 30GHz on a spectrum analyzer. The black trace represents the background signal and the red trace the beating signal. The bias of the detector section was set to -0.2 V. The beating signal is well established above the noise floor up to a frequency of 20 GHz. (b) Heterodyne Beating recorded for a detector section bias of -2.0V. For increasing negaitve bias, the beating signal is only pronounced above background noise levels up to a frequency of 15 GHz. Both results were obtained by using the spectrum analyzer Max-Hold setting and tuning the temperature of the single-mode laser in order to sweep the resulting beating signal position.
